# Unraveling the roles of the reductant and free copper ions in LPMO kinetics

**DOI:** 10.1186/s13068-021-01879-0

**Published:** 2021-01-21

**Authors:** Anton A. Stepnov, Zarah Forsberg, Morten Sørlie, Giang-Son Nguyen, Alexander Wentzel, Åsmund K. Røhr, Vincent G. H. Eijsink

**Affiliations:** 1 Faculty of Chemistry, Biotechnology and Food Science, NMBU-Norwegian University of Life Sciences, Ås, Norway; 2 Department of Biotechnology and Nanomedicine, SINTEF Industry, Trondheim, Norway

**Keywords:** Lytic polysaccharide monooxygenase, AA10, Enzyme kinetics, Hydrogen peroxide, Copper, Ascorbic acid, Gallic acid

## Abstract

**Background:**

Lytic polysaccharide monooxygenases (LPMOs) are monocopper enzymes that catalyze oxidative depolymerization of industrially relevant crystalline polysaccharides, such as cellulose, in a reaction that depends on an electron donor and O_2_ or H_2_O_2_. While it is well known that LPMOs can utilize a wide variety of electron donors, the variation in reported efficiencies of various LPMO-reductant combinations remains largely unexplained.

**Results:**

In this study, we describe a novel two-domain cellulose-active family AA10 LPMO from a marine actinomycete, which we have used to look more closely at the effects of the reductant and copper ions on the LPMO reaction. Our results show that ascorbate-driven LPMO reactions are extremely sensitive to very low amounts (micromolar concentrations) of free copper because reduction of free Cu(II) ions by ascorbic acid leads to formation of H_2_O_2_, which speeds up the LPMO reaction. In contrast, the use of gallic acid yields steady reactions that are almost insensitive to the presence of free copper ions. Various experiments, including dose–response studies with the enzyme, showed that under typically used reaction conditions, the rate of the reaction is limited by LPMO-independent formation of H_2_O_2_ resulting from oxidation of the reductant.

**Conclusion:**

The strong impact of low amounts of free copper on LPMO reactions with ascorbic acid and O_2_, i.e. the most commonly used conditions when assessing LPMO activity, likely explains reported variations in LPMO rates. The observed differences between ascorbic acid and gallic acid show a way of making LPMO reactions less copper-dependent and illustrate that reductant effects on LPMO action need to be interpreted with great caution. In clean reactions, with minimized generation of H_2_O_2_, the (O_2_-driven) LPMO reaction is exceedingly slow, compared to the much faster peroxygenase reaction that occurs when adding H_2_O_2_.

## Background

Lytic polysaccharide monooxygenases (LPMOs) are monocopper enzymes that catalyze oxidative cleavage of polysaccharide substrates, such as chitin and cellulose [1–4]. The LPMO active site is formed by two conserved histidine residues coordinating a copper ion in a rare structural motif that is called the “histidine brace” [3, 5]. The histidine brace is part of a solvent-exposed substrate binding surface, which, for LPMOs acting on chitin and cellulose, is characteristically flat [5, 6]. This spatial configuration reflects the unparalleled ability of LPMOs to act on highly crystalline substrate surfaces, rather than on isolated polysaccharide chains within amorphous regions. By doing so, LPMOs provide a substantial boost to conventional hydrolytic enzymes both in nature and in commercial enzyme cocktails [7, 8]. Due to their intriguing capabilities and industrial applications, there is considerable interest in discovering new LPMOs and in understanding how to optimally harness the catalytic potential of these enzymes.

LPMOs rely on reducing power to enable the formation of reactive oxygen species that hydroxylate glycosidic bonds at the C1- or C4-position [1, 4, 9]. Interestingly, LPMOs can utilize a vast variety of electron donors, including small phenolic compounds and partner enzymes such as cellobiose dehydrogenase [10, 11]. Ascorbic acid is typically used as a reductant in most experimental setups. LPMO reactions were previously thought to involve molecular oxygen, hence the name monooxygenase, but recently, it has been shown that hydrogen peroxide is the preferred co-substrate [12]. H_2_O_2_-driven reactions are orders of magnitude faster than the reactions with oxygen, where the latter tend to be slow, with rates normally being around 1 min^−1^ or lower. Importantly, the supply of H_2_O_2_ needs to be controlled to avoid enzyme damage due to self-oxidation [12].

In the presence of oxygen, reduced LPMOs are capable of H_2_O_2_ production. This phenomenon was initially described as a futile reaction that occurs in the absence of a substrate [13]. Given the current insights into the role of H_2_O_2_ as the (preferred) co-substrate, it has been suggested that under commonly used standard aerobic conditions, i.e., in the presence of a reductant and with no exogenously added hydrogen peroxide, the rate and yield of the LPMO reaction are determined by the in situ generation of H_2_O_2_ that is produced by the enzyme or in reactions involving the reductant and molecular oxygen [12]. It is worth noting that there is an ongoing debate on whether truly O_2_-driven LPMO reactions (i.e., reactions that are not coupled to in situ H_2_O_2_ production) can occur at all [14–17].

Importantly, enzyme-independent H_2_O_2_ production can take place in typical LPMO reaction setups, especially if the reaction, next to a reductant such as ascorbic acid, also contains free transition metal ions, such as Cu(II) [18, 19]. Such enzyme-independent generation of hydrogen peroxide could lead to a substantial boost of LPMO activity on polysaccharide substrates [20]. Due to the use of different enzyme preparation methods and/or reaction conditions, the free copper content of LPMO reactions may vary, which, considering the above, will have repercussions for the reliability and comparability of observed LPMO activities. For example, a significant amount of metal ions may enter the reaction if a contaminated substrate is used, or in case the target enzyme is not sufficiently purified after copper saturation [21].

In this paper, we describe a cellulose-active family 10 (AA10) LPMO, AA10_07, that was discovered by mining the genome of a marine Actinomycete, isolated from the Trondheim fjord, Norway and referred to as “strain P01-F09” below. Next to characterizing the activity of this LPMO, we have used this enzyme as a model to study how reductants and free copper affect LPMO activity on cellulose and to study if and how the observed catalytic activity can be linked to production of H_2_O_2_ in the reaction mixture. Our results demonstrate that ascorbate-driven LPMO reactions are extremely sensitive to free copper in micromolar concentrations, whereas use of gallic acid as reductant allows for steady and controllable reactions that are almost insensitive to the presence of free copper ions.

## Results and discussion

### Identification, sequence and domain structure of AA10_07

**Fig. 1:**
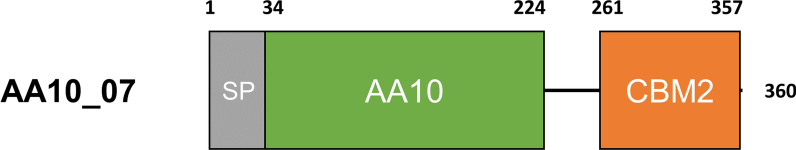
Domain architecture of AA10_07. *SP*, signal peptide; *AA10*, catalytic domain; *CBM2*, family 2 cellulose-binding module. Domain boundaries were annotated using Pfam [22]. Note that the native signal peptide (shown in the figure) was substituted by the pelB signal peptide in the enzyme produced in this study, and that the signal peptide is cleaved off during secretion resulting in the mature enzyme that starts at position 34 (His34)

Actinomycete strain P01-F09 was isolated from a finger sponge harvested at 60 m depth near Tautra, an island located within the Trondheim fjord, Norway. In silico mining of the P01-F09 draft genome sequence using LPMO HMM profiles led to the identification of a 1083 bp gene encoding a hypothetical family 10 (AA10) LPMO. The candidate enzyme was named AA10_07 and its sequence was annotated using the Pfam domain prediction server [22]. AA10_07 is a 360-residue protein, comprising a 33-residue signal peptide, a catalytic AA10 domain and a C-terminal cellulose-binding module (CBM2) (Fig. 1). The two AA10_07 domains are connected through a linker rich in proline and threonine. BLAST analysis [23] identified *Sc*LPMO10C (CelS2) [2, 24] as the closest characterised AA10_07 homolog (85.6% sequence identity between catalytic domains).

### Enzyme production and characterization of LPMO activity

The AA10_07 gene sequence was codon optimized for expression in *E. coli* and modified to encode the pelB periplasmic localization signal [25, 26] instead of the native signal peptide. The gene was cloned into the pET-26(b)+ vector and the enzyme was produced in *E. coli* BL21(DE3) in a soluble form. Starting with a periplasmic extract, AA10_07 was purified to electrophoretic homogeneity by ion-exchange and size-exclusion chromatography. The final yield amounted to approximately 7 mg of purified LPMO per 500 ml of *E. coli* culture.

**Fig. 2:**
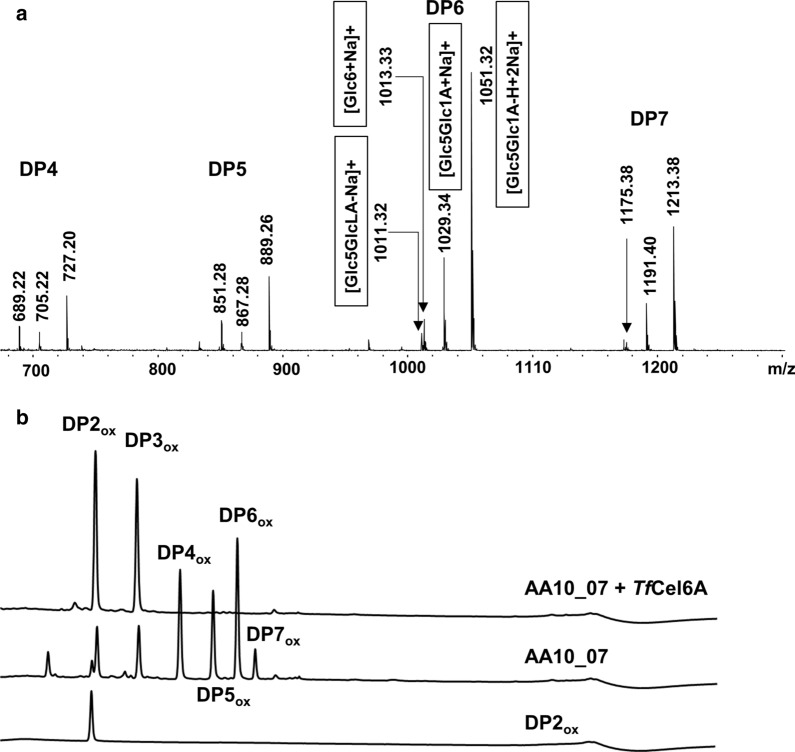
Catalytic activity of AA10_07. **a** MALDI-TOF MS analysis of products released by 1 µM AA10_07 from 1% (w/v) Avicel in the presence of 1 mM ascorbic acid. The figure shows signals representing LPMO products within the DP4–DP7 range (*DP*, degree of polymerization). Oxidized and native products are observed as sodium adducts and/or as sodium adducts of sodium salts, as indicated (*Glc*, glucose; *Glc1A*, gluconic acid; *GlcLA*, lactone). Annotated product peaks were not detected in negative control reactions lacking the enzyme or lacking the reductant. **b** A chromatographic analysis of oxidized products generated by 1 µM AA10_07 from 1% (w/v) Avicel. The picture includes a chromatogram of a product mixture that had been treated with TfCel6A and a chromatogram showing the GlcGlc1A (DP2ox) standard

To assess AA10_07 activity, 1 µM copper-saturated enzyme was incubated with 1% (w/v) Avicel in 50 mM sodium phosphate buffer, pH 6.0, supplied with 1 mM ascorbic acid (30 °C, 24 h). The MS spectrum of the reaction mixture (Fig. 2a) shows a product profile that is typical for LPMOs that exclusively oxidize cellulose at C1. Such oxidation leads to the formation of aldonic acids that give characteristic MS signals due to the formation of sodium and potassium salts. We did not detect any signals that could indicated the formation of double oxidized products, which would appear, albeit at low intensities, if C4 oxidation would also have occurred [24]. This oxidative regioselectivity of the LPMO reaction was confirmed by chromatographic analysis of products generated from Avicel, which only showed C1-oxidized products (Fig. 2b). Figure 2b also shows how the product profile changes after treating the products with *Tf*Cel6A endoglucanase; this approach was used for quantification of oxidized sites in the experiments described below.

### The impact of the copper saturation protocol on the level of residual free copper in LPMO samples

**Fig. 3:**
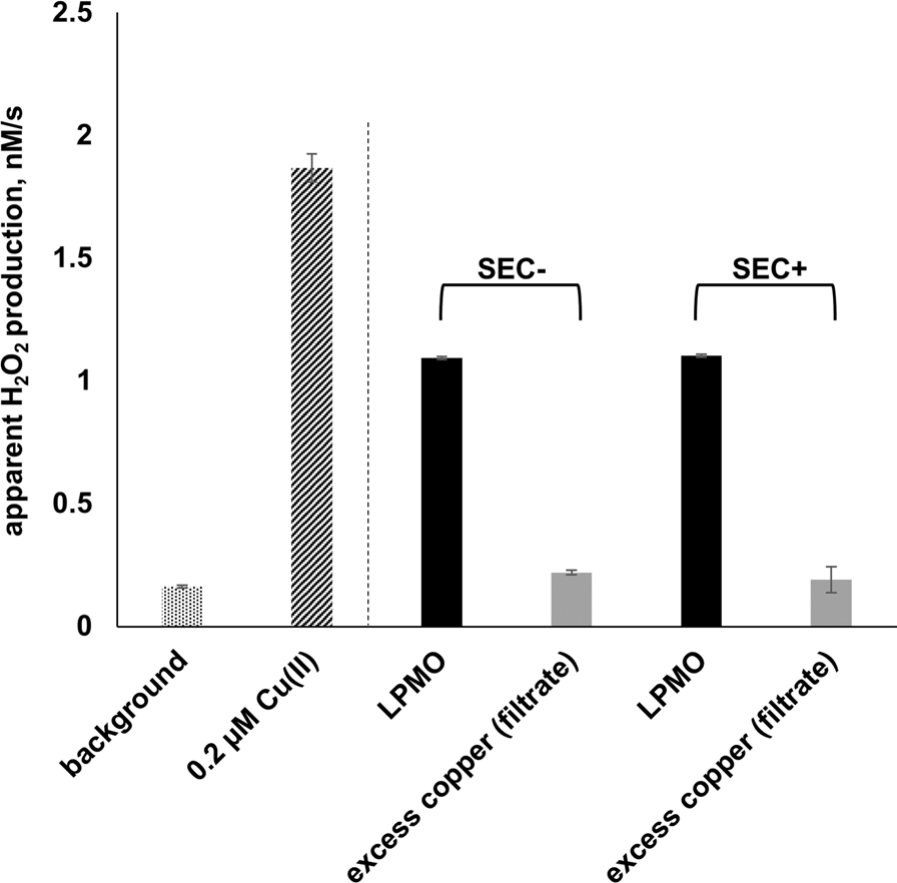
Apparent hydrogen peroxide production by two batches of AA10_07. The figure shows apparent H_2_O_2_ production by 3 µM AA10_07 in 50 mM sodium phosphate buffer, pH 6.0 supplied with 50 µM ascorbic acid, 5 U/ml HRP, 100 µM Amplex Red and 1% (v/v) DMSO. SEC−/SEC+ labels indicate the procedure used to remove unbound copper from the LPMO sample after copper saturation. SEC−, gravity-flow desalting column; SEC+, high-resolution SEC chromatography. Excess copper control reactions (grey bars) were set up using protein-free samples, obtained by ultrafiltration. These samples contained the same amount of free copper as the LPMO preparation used in the experiment. The two reactions shown to the left are control reactions: “background”, reaction without enzyme; “0.2 µM Cu(II)”, reaction without enzyme and with addition of 0.2 µM Cu(II). Error bars indicate standard deviations between triplicates

Free copper ions will affect LPMO reactions since they promote enzyme-independent H_2_O_2_ production in the presence of ascorbic acid, even when present at sub-micromolar concentrations ([19–21]; see also Fig. 3). It is reasonable to assume that LPMO preparations vary in terms of the amounts of free copper, due to variations in protein preparation protocols, and this may affect the apparent activity of the LPMOs in certain reaction setups. Copper saturation of the purified LPMO likely is the most critical step, since during that stage a free copper is deliberately introduced into the system.

Here, and in several other studies, copper saturation was achieved by incubating the LPMO with a slight molar surplus of free copper, followed by desalting. We compared AA10_7 samples obtained by two alternative desalting techniques. In one case, unbound copper was removed by fast desalting using a small gravity-flow gel filtration column. In the other case, a high-resolution preparative SEC column was used instead. The resulting two different batches of copper saturated and desalted AA10_07 showed identical apparent rates of H_2_O_2_ production (Fig. 3) and identical abilities to degrade Avicel (Additional file 1: Fig S1), in reactions with ascorbic acid as the reductant. These observations indicate that both procedures worked equally well in terms of removing free copper.

The copper content of the resulting enzyme preparations was assessed in two manners. Protein-free fractions (“filtrates”) produced from these LPMO samples by ultrafiltration did not promote H_2_O_2_ production in a reaction with ascorbic acid, whereas added copper in concentrations as low as 0.2 μM did (Fig. 3), indicating that the levels of residual copper were low in both enzyme samples. To confirm this conclusion, both LPMO samples were subjected to ICP-MS analysis. The results indicated the presence of 0.98 ± 0.18 µM and 0.90 ± 0.14 µM total copper in 1 µM solutions of gravity flow-desalted and SEC-treated LPMO, respectively. These ICP-MS results are compatible with the notion that the LPMOs were copper saturated, whereas the amounts of free copper were negligible. Finally, the copper content of Avicel was determined by ICP-MS showing that only negligible amounts of this metal were present (< 49 ng copper per 1 g of substrate, corresponding to less than 10 nM in reactions with 1% (w/v) Avicel).

### Comparison of ascorbic acid and gallic acid in LPMO reactions with Avicel

**Fig. 4:**
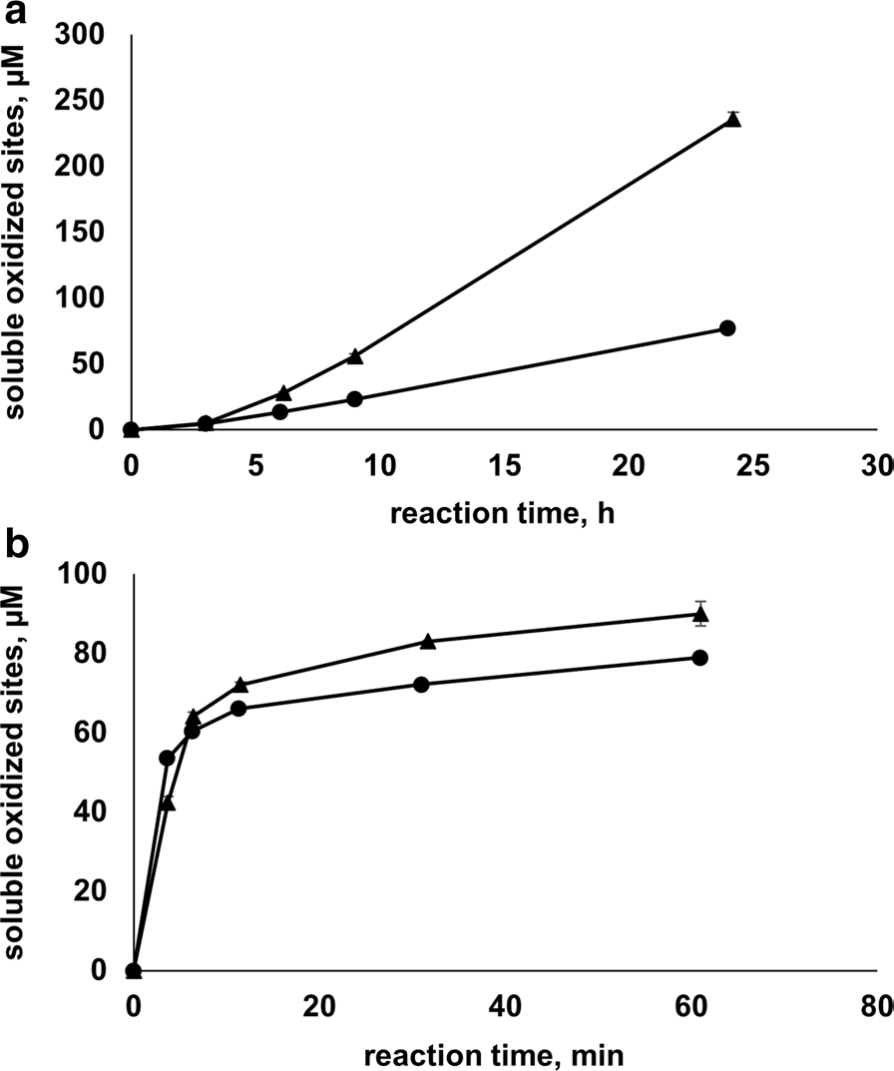
Cellulose solubilisation by AA10_07 in the presence of ascorbic acid or gallic acid. The figure shows the release of oxidised products in LPMO reactions (1 µM AA10_07 in 50 mM sodium phosphate buffer, pH 6.0, 30 °C) with 1% (w/v) Avicel that were carried out in the absence of hydrogen peroxide (**a**) or in the presence of 200 µM H_2_O_2_ (**b**) using 1 mM ascorbic acid (circular markers) or 1 mM gallic acid (triangle markers) as a reductant. Error bars indicate standard deviations between triplicates. Note that **a**, **b** feature different time scales. Product accumulation was not observed in control reactions with substrate and reductant or in reactions with substrate, reductant and H_2_O_2_

Next, we assessed the capacity of gallic acid to drive the oxidation of cellulose by AA10_07. The LPMO reaction supplied with 1 mM gallic acid was more efficient than the reaction supplied with 1 mM ascorbic acid (Fig. 4a). Both reactions were remarkably slow (approximately 0.06 min^−1^ in the presence of ascorbic acid and 0.2 min^−1^ in the presence of gallic acid) with almost linear progress within the 24 h of the experiment.

To verify that the LPMO was catalytically competent and that H_2_O_2_ speeds up the reaction, a control experiment with externally added H_2_O_2_ was conducted, which showed an increase in the initial substrate oxidation rate by two orders of magnitude and, as expected under these conditions, rapid inactivation, regardless of whether the reductant was ascorbic acid or gallic acid (Fig. 4b; note that the *X*-axis has a minutes time scale).

### Apparent H_2_O_2_ production in reactions with LPMO, free copper and 50 µM reductant

**Fig. 5:**
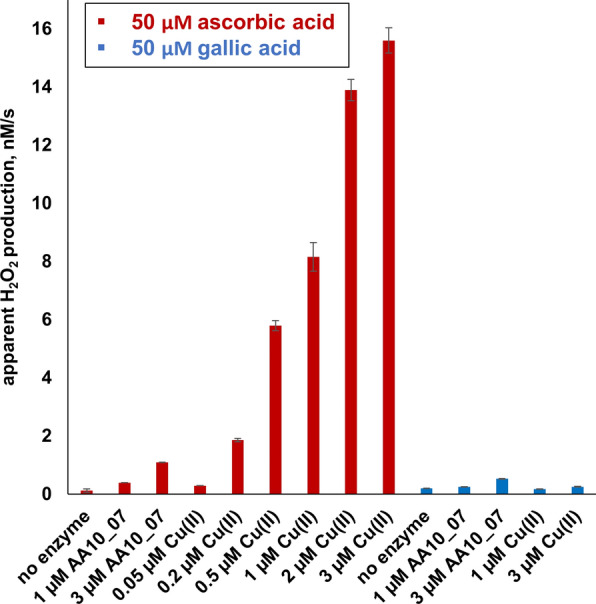
Apparent H_2_O_2_ production in reactions with free copper and 50 µM ascorbic acid or gallic acid. All experiments were carried out in 50 mM sodium phosphate buffer, pH 6.0, at 30 °C. The rates of H_2_O_2_ production by AA10_07 in the presence of 50 µM ascorbic acid or 50 µM gallic acid are given for reference. The experiment with 0.2 µM Cu(II) is also shown in Fig. 3. Reaction mixtures contained 5 U/ml HRP, 100 µM Amplex Red and approximately 1% (v/v) DMSO. Error bars indicate standard deviations between triplicates

To further investigate the effects of free copper, we assessed H_2_O_2_ production in a series of reactions with various amounts of Cu(II)SO_4_ in the presence of two commonly used LPMO reductants, ascorbic acid and gallic acid. Of note, to reduce complications due to reactions between HRP and the reductant (see below), the reductant concentrations typically used in the HRP/Amplex Red H_2_O_2_ assay are much lower (e.g. 50 μM; [13, 27]) than those typically used in LPMO reactions with substrate (e.g. 1 mM). The results of the standard H_2_O_2_ assay (Fig. 5) show that sub-micromolar concentrations of free copper increased the apparent rate of H_2_O_2_ production in reactions with 50 µM ascorbic acid. Even the reaction with only 0.2 µM free copper gave a higher apparent H_2_O_2_ production rate than a reaction lacking free copper but containing 3 µM copper-loaded AA10_7. It is noteworthy that these results may be taken to suggest that the LPMO protects reduced copper from reacting with molecular oxygen. Similar experiments with 50 µM gallic acid showed different results. The apparent initial rate of H_2_O_2_ formation by 3 µM AA10_07 in the presence of 50 µM gallic acid amounted to 0.53 nM/s, which is two times lower compared to the reaction with ascorbic acid (Fig. 5). Strikingly, hydrogen peroxide accumulation in the presence of free copper and gallic acid was very low compared to similar reactions with ascorbate (Fig. 5). For example, in large contrast to the results obtained with ascorbic acid, the apparent H_2_O_2_ production rate in the reaction with 3 µM free copper and gallic acid was not higher than the one observed with LPMO and gallic acid under the same conditions. The observed low reactivity of free copper in the presence of gallic acid is in agreement with electron paramagnetic resonance data [28] showing that gallic acid is likely to form complexes with Cu(II) rather than reducing it.

### Apparent H_2_O_2_ production in the presence of 1 mM reductant

The experiments with AA10_07 and gallic acid revealed lower apparent H_2_O_2_ production rates compared to the similar setups with ascorbate. However, the LPMO catalytic rate on Avicel was significantly higher in the presence of gallic acid, which is surprising if one accepts the premise that generation of H_2_O_2_ limits the reaction.

In search of an explanation for this paradoxical finding, it is important to consider the limitations of the HRP/Amplex Red assay [29]. The assay is based on single electron oxidation of Amplex Red by HRP in the presence of H_2_O_2_ as a co-substrate [30]. This oxidation leads to the formation of two Amplex Red radicals, which then react to form one molecule of highly chromogenic resorufin and one molecule of Amplex Red. It was previously shown that addition of ascorbic acid leads to repression of the resorufin signal [15], likely due to reduction of Amplex Red radicals back to Amplex Red [30] and/or the ability of HRP to engage in a side-reaction with ascorbate that removes H_2_O_2_ from the system in a way that is uncoupled to resorufin formation [31]. There is evidence that other redox-active compounds may also influence the HRP/Amplex Red assay in a similar fashion [32, 33]. It is thus conceivable that the different apparent H_2_O_2_ production rates for ascorbic acid and gallic acid relate to varying degrees of signal repression. To test this hypothesis, we carried out the HRP/Amplex Red assay using different concentrations of H_2_O_2_ in the presence or absence of 50 µM ascorbic acid or gallic acid. This experiment indicated that gallic acid supresses the resorufin signal to approximately same extent as ascorbic acid (Additional file 1: Fig. S2).

We then asked the question whether the discrepancy between apparent H_2_O_2_ production rates and the observed LPMO activities in reactions with Avicel could be due to the largely different reductant concentrations in these two experiments. While low reductant concentrations are commonly used in the H_2_O_2_ production assay, for reasons discussed above, experiments with 1 mM reductant are rare [34]. Thus, we carried out the HRP/Amplex Red assay using various concentrations of hydrogen peroxide in the presence of 1 mM ascorbic acid or gallic acid, which showed that the resorufin signal repression increased only marginally compared to the previous measurement with 50 µM reductant (Additional file 1: Fig S2). Thus, measurements of H_2_O_2_ production in reactions with 1 mM reductant seemed feasible.

**Fig. 6:**
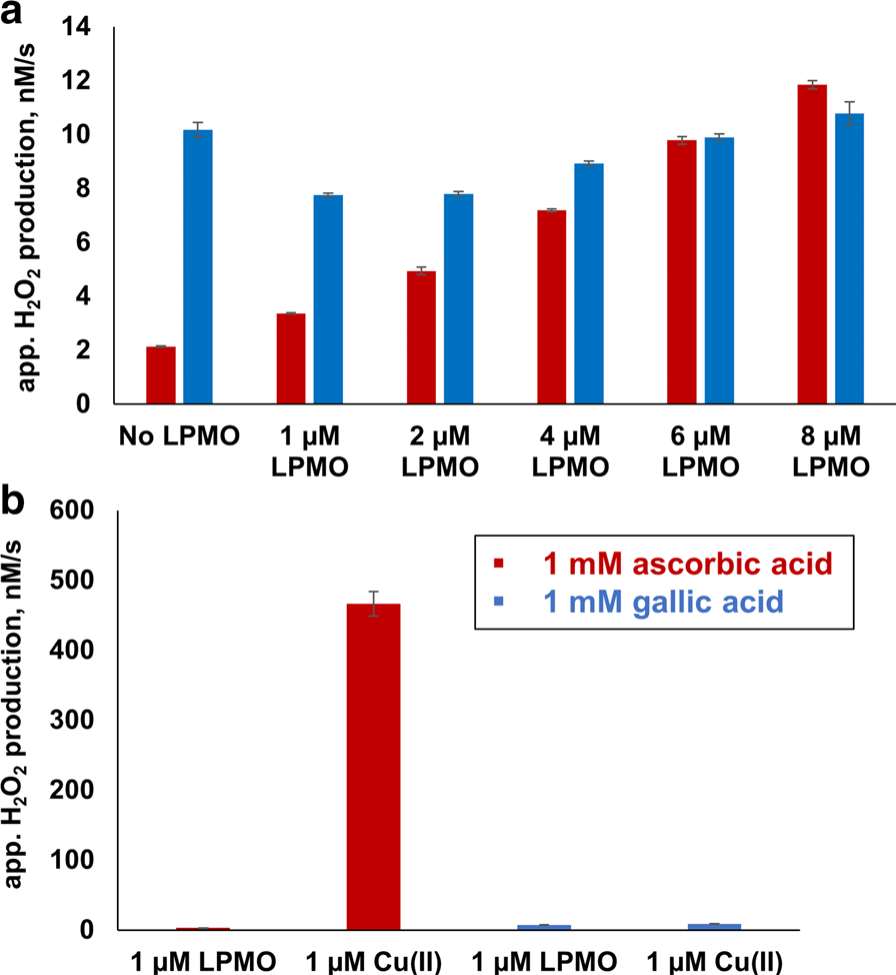
Apparent H_2_O_2_ production in experiments with LPMO, free copper and 1 mM ascorbic acid or gallic acid. The figure shows hydrogen peroxide accumulation rates obtained in reactions with increasing amounts of LPMO (**a**) or with 1 µM free copper compared to 1 µM LPMO (**b**). All experiments were carried out in 50 mM sodium phosphate buffer, pH 6.0, at 30 °C. Reaction mixtures contained 5 U/ml HRP, 100 µM Amplex Red and 2% (v/v) DMSO. Error bars indicate standard deviations between triplicates. Note that plotted values were corrected for resorufin signal repression using H_2_O_2_ standard curves, obtained in the presence of 1 mM ascorbic acid or gallic acid. The “No LPMO” label indicates that the reaction contained only reductant, with no enzyme added (set up to monitor reactions between reductant and oxygen)

Therefore, the HRP/Amplex Red assay was performed again in the presence of 1 mM ascorbic acid or 1 mM gallic acid, together with various amounts of LPMO and free copper (Fig. 6a, b). Apparent hydrogen peroxide accumulation rates were corrected for resorufin signal repression using H_2_O_2_ standard curves obtained in the presence of reductants. In this setup, the H_2_O_2_ production rate by 1 µM AA10_07 was approximately 2.3 times higher with gallic acid compared to ascorbic acid. Assuming that generation of H_2_O_2_ limits the reaction, the higher H_2_O_2_ production level observed in the reaction with 1 mM gallic acid is in agreement with Avicel degradation data (Fig. 4a) showing that 1 µM AA10_07 was most efficient when using gallic acid as reductant. Quantitative comparison of the Avicel degradation data (Fig. 4a) and the H_2_O_2_ production data (Fig. 6a) indicates that the production rate of soluble oxidized products is roughly half of the H_2_O_2_ production rate, which makes sense considering that a considerable fraction of the oxidized sites will remain attached to the insoluble substrate.

Strikingly, when using 1 mM ascorbic acid concentration, H_2_O_2_ production by 1 µM free copper was two orders of magnitude higher compared to 1 µM LPMO, whereas H_2_O_2_ production in the reaction with free copper and gallic acid was minimal, also at this higher reductant concentration (Fig. 6b). Clearly, in standard LPMO reactions with 1 mM ascorbic acid, the effect of free copper may be considerable, whereas such a copper effect could be less or even absent in reactions with 1 mM gallic acid. This is addressed further below.

One key question is to what extent the enzyme plays a role in the generation of hydrogen peroxide in the LPMO reaction. Figure 6a shows that the H_2_O_2_ accumulation rates depended on the enzyme concentration, and that this dependency was stronger for ascorbic acid than for gallic acid. An eightfold increase in LPMO concentration (from 1 to 8 µM) resulted in 3.5 times higher H_2_O_2_ accumulation rates for ascorbic acid, whereas the increase was only a modest and 1.4-fold for gallic acid. In the case of ascorbic acid, the reaction with the lowest LPMO concentration (1 μM) generated more H_2_O_2_ than a reaction without enzyme. In contrast, for gallic acid, the experiment without enzyme gave higher hydrogen peroxide levels than the experiments with 1–6 µM enzyme, which may indicate that, in the absence of cellulose substrate, AA10_07 engages in side-reactions that consume H_2_O_2_ and involve gallic acid (or products of gallic acid oxidation). Such reactions are not entirely hypothetical, since it is well known that LPMOs can carry out peroxygenation reactions of small molecules. Breslmayr et al*.* have shown that after being reduced by 2,6-dimethoxyphenol, LPMOs can oxidize the hydrocoerulignone that is formed by dimerization of two 2,6-dimethoxyphenol radicals in an (H_2_O_2_-consuming) peroxygenation reaction [35]. Similar reactions could occur with dimers of gallic acid that are likely to emerge upon its oxidation [36].

Overall, our results suggest that the amount of hydrogen peroxide produced by 1 µM LPMO supplied with 1 mM gallic acid is low (Fig. 6a), compared to the amount of H_2_O_2_ generated by the enzyme-independent reaction between reductant and oxygen. In other words, the auto-oxidation of 1 mM gallic acid is likely to fuel LPMO reactions on Avicel in our setups. In the case of ascorbic acid, both the LPMO and the reductant contribute to generation of H_2_O_2_, at least in the absence of an LPMO substrate (see below).

### Effect of the LPMO concentration on degradation of Avicel

**Fig. 7:**
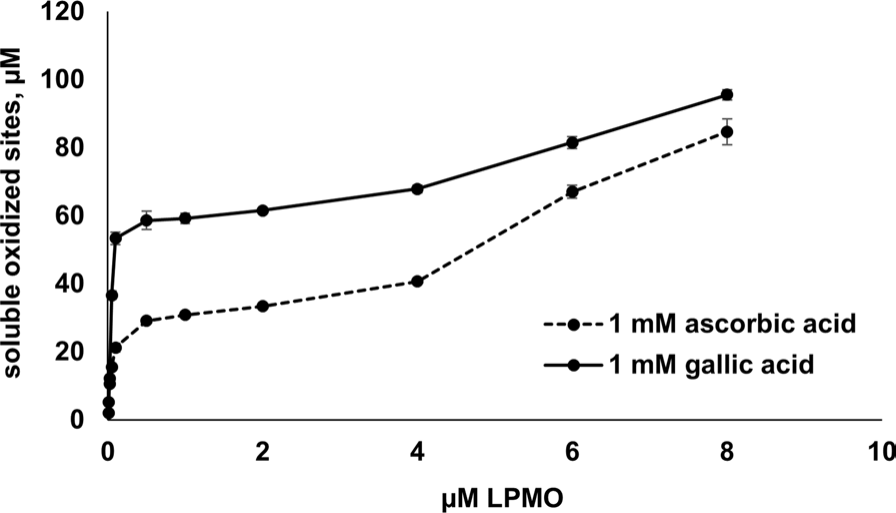
Avicel solubilisation by increasing amounts of AA10_07 in the presence of ascorbic acid or gallic acid. The figure shows the release of oxidised products from 1% (w/v) Avicel in 9 h reactions with 0.01–8 µM AA10_07 in 50 mM sodium phosphate buffer, pH 6.0, 30 °C. The experiments were carried out using 1 mM ascorbic acid (dashed line) or 1 mM gallic acid (solid line) as a reductant. Error bars indicate standard deviations between triplicates. Note that the plotted data are derived from LPMO progress curves shown in Additional file 1: Fig. S3. All these reactions displayed linear behavior, except for experiments with 0.01 µM or 0.025 µM enzyme and gallic acid

To obtain further insight into factors that limit the LPMO reaction under typical LPMO assay conditions, without depending on the HRP/Amplex Red assay, we carried out a series of LPMO reactions with Avicel (Fig. 7, Additional file 1: Fig. S3) in the presence of 1 mM ascorbic acid or 1 mM gallic acid using a wide range of AA10_07 concentrations (0.01–8 µM). In light of the H_2_O_2_ accumulation data shown in Fig. 6a, and assuming that enzyme-independent generation of H_2_O_2_ limits the reaction, one could expect limited effects of the LPMO concentration on the cellulose degradation rate, in particular for reactions with gallic acid.

Such behaviour was indeed observed in the experiment with 1 mM gallic acid, (Fig. 7, Additional file 1: Fig. S3). Within the enzyme concentration range of 0.1 µM to 4 µM, the enzyme dose had almost no effect on the rate of cellulose degradation. Note that progress curves (Additional file 1: Fig. S3) obtained for the reactions with 0.1–4 µM LPMO were linear, showing that the amount of substrate was not limiting these reactions.

Further increase of the LPMO dose resulted in a gradual increase in the rate of substrate oxidation, indicating that the enzyme’s contribution to H_2_O_2_ production becomes significant at higher enzyme concentrations. At very low enzyme concentrations (< 0.1 µM), there was a strong dose-dependency, most likely due to the fact that there was not enough enzyme to consume H_2_O_2_ effectively (i.e., the reaction was limited by the enzyme and not by hydrogen peroxide). The experiment with 1 mM ascorbic acid revealed the same low dependency of the cellulose oxidation rate on the LPMO concentration in the 0.1–4 µM range (Fig. 7, Additional file 1: Fig. S3). This observation was unexpected if one considers the H_2_O_2_ accumulation data (Fig. 6a), which suggest that, in reactions with ascorbic acid, the LPMO contribution to overall hydrogen peroxide generation is considerable. One obvious, but nevertheless speculative, explanation would be that binding of the LPMO to the substrate reduce the enzyme’s ability to generate H_2_O_2_ [13, 15, 37]. Interestingly, at the higher LPMO concentrations, there was a dose–response effect that was more pronounced compared to the similar reactions with gallic acid, which is in agreement with the H_2_O_2_ accumulation data of Fig. 6a. Overall, our results indicate that under most commonly used reaction conditions, oxidation of the reductant by O_2_ is a major source of H_2_O_2_ that fuels cellulose degradation by AA10_07.

### The effect of free copper on LPMO reactions with Avicel

**Fig. 8:**
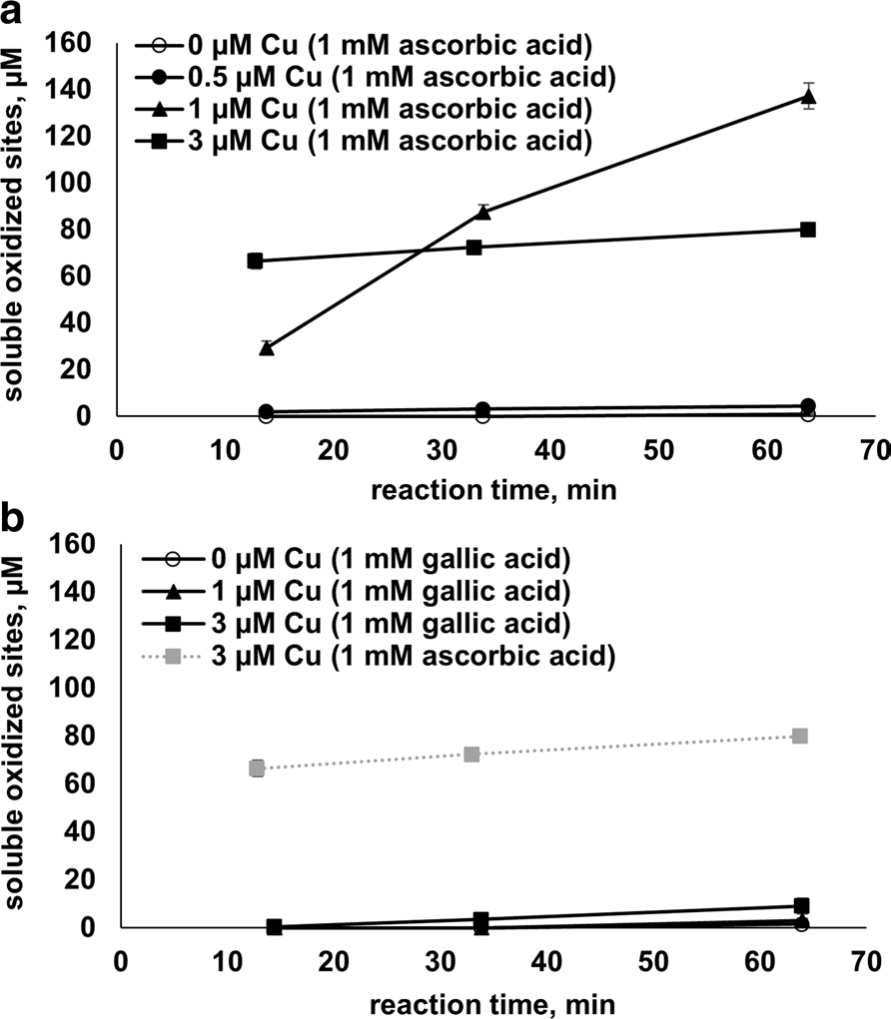
The effect of free copper on AA10_07 reactions with cellulose in the presence of ascorbic acid or gallic acid. The figure shows progress curves obtained for AA10_07 reactions (1 µM LPMO in 50 mM sodium phosphate buffer, pH 6.0, 30 °C) with 1% (w/v) Avicel using 1 mM ascorbic acid (**a**) or 1 mM gallic acid (**b**) and various concentrations of free copper. Note that **b** also features the progress curve obtained with 3 µM free copper and 1 mM ascorbic acid, which is shown in **a**, for reference. Error bars indicate standard deviations between triplicates. Note the different time scale compared to Fig. 4a. Product accumulation was not observed in control reactions with no enzyme, containing substrate, free copper, and reductant

Digging further into interactions between enzyme, free copper and electron donors, we carried out degradation reactions with Avicel using 1 mM ascorbic acid or 1 mM gallic acid as the reductant. Various amounts of free copper were added to the reactions to mimic copper contamination and to vary the rate of enzyme-independent production of H_2_O_2_. The addition of Cu(II) to reactions with ascorbic acid resulted in a copper dose-dependent increase in LPMO activity (Fig. 8a), which is to be expected considering the high rates of H_2_O_2_ production in reactions with free copper and 1 mM ascorbic acid (Fig. 6b). Comparison of Figs. 4a and 8a shows that a 1 h reaction in the presence of 1 µM Cu(II)SO_4_ and ascorbic acid (Fig. 8a) yielded more oxidized products than a 24 h copper-free reaction with the same reductant (Fig. 4a). At 3 μM copper, the reaction is even faster, leading to high product concentrations at the first measuring point, but under these conditions, the enzyme becomes rapidly inactivated, as one would expect if (too) much H_2_O_2_ is produced [12, 20].

On the other hand, the LPMO reaction driven by gallic acid (Fig. 8b) turned out to be essentially insensitive to free copper (up to 3 µM Cu(II)SO_4_), which is in agreement with the H_2_O_2_ production data (Figs. 5, 6) and with the notion that this reductant forms complexes with Cu(II), thus preventing Cu(II) from participating in redox reaction in solution [28].

Estimating catalytic rates from the progress curves of Figs. 4a, 8a shows that introduction of 1 µM Cu(II) to the LPMO reaction fuelled by ascorbate speeded up the reaction by some 50-fold, which is in the same order of magnitude as the effect of 1 µM Cu(II) on ascorbic acid-driven production of H_2_O_2_ (Fig. 6b). Nevertheless, the LPMO reaction fuelled by ascorbate and in the presence of 1 µM Cu(II) was still much slower and showed less rapid enzyme inactivation (Fig. 8a) compared to the reaction with initial addition of 200 µM hydrogen peroxide (Fig. 4b). This observation is not surprising, considering the hydrogen peroxide production data of Fig. 6b that show an approximate H_2_O_2_ production rate of 0.5 µM/s for the reaction with 1 µM Cu(II) and 1 mM ascorbic acid. Clearly, despite the effect of free copper, the H_2_O_2_ concentration in the reaction with exogenously added H_2_O_2_ was much higher.

The lack of an effect of low copper concentrations (< 1 μM) on the ascorbic acid-driven reaction was somewhat unexpected considering the H_2_O_2_ production curves shown in Fig. 5. It is conceivable that the Avicel used in the experiment possesses a weak copper-binding capacity that removes a fraction of free copper from the reaction. It is also possible that the LPMO has secondary (low affinity) copper-binding sites [38]. Finally, it is possible that under the conditions of the Avicel assay, copper and/or H_2_O_2_ engage in side-reactions that may reduce H_2_O_2_ production or increase futile H_2_O_2_ consumption, hiding the copper effect on LPMO activity at the lowest copper concentrations.

The sensitivity of the LPMO reaction to free copper in the presence of ascorbic acid must be taken into account when interpreting previously obtained dose–response curves for AA10_07 (Fig. 7). It is conceivable that the dependency of the product formation rate on the amount of LPMO observed at high enzyme concentrations (> 4 µM) is explained by increasing amounts of free copper that are introduced to the reaction together with the enzyme. A control experiment with protein-free ultrafiltrates of the enzyme stock solutions showed that this was not the case (Additional file 1: Fig. S4).

### Copper bias in previously reported LPMO data

The Avicel degradation experiments with either ascorbic acid or gallic acid (in the absence of exogenous H_2_O_2_ and free copper) indicate that AA10_07 is a slow enzyme. Early in the reaction, i.e., within the first hour or so, the release of soluble products is close to negligible (Fig. 4a), which is likely due to the fact that in this early phase of the reaction, oxidized products are still polymeric and, thus, not soluble. Frommhagen et al. [39] have pointed out that it takes a while before the same cellulose chain has been cleaved sufficiently many times to generate short, soluble products that are released into solution. Later during the reaction (Fig. 4a), the release of oxidized products over time was close to linear, but very slow, with rates (estimated from Fig. 4a) in the range of 0.06–0.2 min^−1^. Such rates are common among reported rate estimates for LPMOs [40], but these rates are really low and two orders of magnitude lower than rates obtained in reactions with Avicel that are supplied with exogenous H_2_O_2_, which are higher than 10 min^−1^ (Fig. 4b).

AA10_07 is a close homolog of the well-studied *Sc*LPMO10C (CelS2; 85.6% sequence identity between catalytic domains). In previous studies by our group, *Sc*LPMO10C displayed low cellulose oxidation rates (0.1–0.2 min^−1^) in reactions fuelled by ascorbic acid [24, 41], which are comparable to the results obtained here for AA10_07. However, looking back on a larger set of (our own) data, we noted that much higher oxidation rates were observed in other studies while using seemingly comparable conditions, as shown in Table 1. Importantly, all *Sc*LPMO10C experiments quoted in Table 1 were based upon similar LPMO production, purification and copper saturation procedures.

**Table 1:** Cellulose oxidation rates derived from previously reported *Sc*LPMO10C reactions driven by ascorbic acid and molecular oxygen

Approximate cellulose oxidation rate	Product quantification routine^c^	Substrate and reaction conditions	References
0.2 min^−1 a^	DP2ox quantification (HPAEC-PAD) after treatment with *Tr*Cel7A cellobiohydrolase	0.2% (w/v) PASC, 20 mM ammonium acetate buffer, pH 6.0, 40 °C, 2 mM ascorbic acid	[24]: Fig. 2a
0.14 min^−1 a^	DP2ox quantification (HPAEC-PAD) after treatment with TfCel5A endogluconase	1% (w/v) Avicel, 50 mM sodium phosphate buffer, pH 6.0, 40 °C, 1 mM ascorbic acid	[41]: Fig. 2
6.7 min^−1^	DP2ox + DP3ox quantification (HPAEC-PAD) after treatment with TfCel5A endogluconase	1% (w/v) Avicel, 50 mM sodium phosphate buffer, pH 7.0, 40 °C, 1 mM ascorbic acid	[42]: Fig. 4
3.4 min^−1 b^	DP2ox + DP3ox quantification (HPAEC-PAD) after treatment with TfCel5A endogluconase	1% (w/v) Avicel, 50 mM sodium phosphate buffer, pH 7.0, 40 °C, 1 mM ascorbic acid	[12]: Fig. 1f
2.5 min^−1 b^	DP2ox + DP3ox quantification (HPAEC-PAD) after treatment with TfCel5A endogluconase	0.5% (w/v) PASC, 50 mM sodium phosphate buffer, pH 6.0, 40 °C, 1 mM ascorbic acid	[43]: Fig. 8
7.9 min^−1^	DP2ox + DP3ox quantification (HPAEC-PAD) after treatment with *Tf*Cel5A endoglucanase	1% (w/v) Avicel, 50 mM sodium phosphate buffer, pH 7.0, 40 °C, 1 mM ascorbic acid	[20]: Fig. 2a

^a^The reported rate is underestimated by approximately twofold due to limitations in product quantification. Only C1-oxidized cellobiose was quantified in the mixture of C1-oxidized cellobiose and C1-oxidized cellotriose. The molar ratio between these products is typically close to 1 in reactions with *Sc*LPMO10C [41]

^b^No progress curves were reported in the paper meaning that approximate oxidation rates were estimated using single time points

^c^DP2ox/DP3ox: C1-oxidized cellobiose/cellotriose

Given the fact that all these studies involved ascorbic acid as the reductant, it is reasonable to assume that the observed variations in LPMO activity were caused by different levels of free copper in the reactions (as well as, in some cases, other factors such as pH and the type of substrate).

Comparison of two in-house produced batches of *Sc*LPMO10C, one freshly prepared and one older batch that had shown “high activity”, in the same experiment indeed showed large differences in product yields (Additional file 1: Fig. S5). Most importantly, the difference between the two protein batches disappeared after the most active batch had been subjected to another round of desalting. Quantification of total copper by ICP-MS indicated the presence of 0.9 ± 0.139 µM and 1.7 ± 0.298 µM copper ions in 1 µM enzyme solutions of the freshly prepared and the older “active” enzyme batches, thus confirming that the observed activity differences indeed correlated with varying amounts of free copper.

It is worth noting that both *Sc*LPMO10C batches were prepared in the same manner. In both cases (as well as in the other studies featured in Table 1), a gravity-flow desalting column was used to remove excess copper after copper saturation. It is reasonable to assume that the efficiency of the desalting procedure may have varied. The desalting experiments described above show how LPMO preparations devoid of free copper can be prepared and the filtration experiment depicted in Fig. 3 provides a simple method to check for free copper that does not depend on the use of an ICP-MS.

## Conclusions

Taken together, our results indicate that the use of ascorbic acid as a reductant in LPMO experiments is not optimal, if reproducibility of kinetic data is considered. Ascorbate-driven reactions are sensitive to micromolar concentrations of free copper due to high levels of copper-catalyzed enzyme-independent H_2_O_2_ production. On the other hand, when using gallic acid as an electron donor, the LPMO progress curves show no strong dependency on free copper in the system (at least up to a 3 µM concentration). This is most likely due to complexation of free Cu(II) by gallic acid, which prevents copper reduction.

Potential sources of free copper in LPMO reactions are numerous. LPMO samples may be contaminated as a result of a copper saturation procedure, in case excess Cu(II) ions are not completely removed from the system. Other components of typical LPMO reactions, such as the substrate, may also contain small amounts of copper. Notably, copper carry-over may be facilitated by secondary low affinity binding sites on the enzyme, as observed in some crystal structures [38], and by poly-histidine affinity tags, which are known to possess a high affinity for Cu(II) [44].

Given that ascorbic acid is the most commonly used reductant in the field, it is likely that published LPMO activity data to some extent are biased by the presence of varying and unknown amounts of free copper in the reactions that were conducted. As we illustrate above, this includes some of our own previous work on *Sc*LPMO10C for which we (implicitly) have reported different (higher) catalytic efficiencies which are due to variations in free copper.

The reported sensitivity to free copper is likely not exclusive to LPMO systems that are fuelled by ascorbate and may also apply to reactions with other reductants. For example, it has been established that autoxidation of both l-cysteine and glutathione, compounds that are regularly used as LPMO reductants [1, 11, 45–47], is catalysed by micromolar amounts of free Cu(II) ions in a manner that leads to the production of hydrogen peroxide [48, 49].

On another note, this study presents an illustration of the complex nature and limitations of the HRP/Amplex Red assay (see also [29]). We demonstrate that apparent H_2_O_2_ production rates obtained by this method using low concentrations of reductants (a rather common approach employed in many LPMO studies) may not necessarily describe trends that exist in reactions on cellulose at standard aerobic conditions (i.e., at ≥ 1 mM reductant concentration).

Our data provide insight into multiple mechanisms that may explain why LPMO activity is reductant-dependent. In this paper we show that, in the presence of substrate, hydrogen peroxide generation by 1 µM of a family AA10 LPMO is negligible compared to the amounts of H_2_O_2_ produced by the autoxidation of 1 mM ascorbic acid and, in particular, 1 mM gallic acid.

It is worth noting that LPMOs may significantly vary in hydrogen peroxide production and, thus, some enzymes, for example fungal AA9 type LPMOs, may show less dependency on reductant autoxidation. Furthermore, the oxidation of reductants by oxygen (both in the presence and in absence of free copper) is likely to be affected by pH (these effects are well-described for ascorbate, e.g. [18, 50]), hence the contribution of this process to overall H_2_O_2_ production may depend on reaction conditions.

All in all, this study sheds light on the complex impact of the reductant on LPMO catalysis. Under commonly used reaction conditions, the large surplus of reductant likely ensures that reduction of the LPMO is not rate-limiting, and the rate of the reaction is determined by the generation of H_2_O_2_, which, at least in the case of cellulose-active AA10s, is dominated by the oxidation of the reductant by oxygen.

## Methods

### Materials

Chemicals were obtained from Sigma-Aldrich (St. Louis, MO, USA) unless indicated otherwise. Microcrystalline cellulose used in this study was Avicel PH-101. Amplex Red was obtained from Thermo Fisher Scientific (Waltham, MA, USA). 10 mM Amplex Red stock solutions were prepared in DMSO and stored in light-protected tubes at − 20 °C. Ascorbic acid and gallic acid were stored at − 20 °C as 100 mM stock solutions in metal-free TraceSELECT water (Honeywell, Charlotte, NC, USA) and DMSO, respectively. Horseradish peroxidase type II (HRP) was stored in 50 mM sodium phosphate buffer, pH 6.0 at 4 °C (at 100 U/ml concentration). Tryptone and yeast extract were obtained from Thermo Fisher Scientific (Waltham, MA, USA).

### Identification of the LPMO gene

Strain P01-F09 was isolated from a finger sponge harvested at 60 m depth near Tautra, an island located within the Trondheim fjord, Norway (063° 36′ 53ʺ N, 010° 31′ 22ʺ E) on September 22, 2005. Phylogenetic analysis (16 s rRNA) indicated the closest taxonomic neighbour being *Streptomyces griseolus* (98.8% sequence identity). Shotgun sequencing of the strain's genome was carried out at BaseClear BV (Leiden, Netherlands) using Illumina HiSeq, revealing a genome size of 7,272,225 bp in 910 scaffolds.

Putative LPMO coding sequences within the obtained scaffolds were identified using the *hmmscan* tool from the HMMER software package version 3.1b2 (http://hmmer.org/) [51]. The Hidden Markov Model (HMM) profiles of all LMPO families were extracted from the dbCAN database [52] (version 7 at the time of the analysis), available from the dbCAN website (http://bcb.unl.edu/dbCAN2/). The analysis led to the identification of a seemingly complete gene putatively encoding an AA10 (family 10) LPMO, here referred to as AA10_07. The AA10_07 sequence has been submitted to Genbank under Accession number MT882343.

### Protein expression

The AA10_07-encoding gene (GenBank accession number MT882343) was codon optimized for expression in *Escherichia coli* and synthesized by GenScript (Piscataway, NJ, USA). The resulting synthetic DNA contained a 66 bp leader sequence, encoding for the pelB signal peptide, followed by the *LPMO* gene (residue 34–360). The gene construct was cloned into the pET-26(b)+ expression vector (Merck, Darmstadt, Germany) using NdeI/XhoI restriction sites and sequenced (Sanger sequencing) by GenScript (Piscataway, NJ, USA).

An AA10_07 expression strain was established by heat-shock transformation of BL21 (DE3) competent cells (Invitrogen, Carlsbad, USA) with the expression vector, according to the supplier’s protocol. The transformed cells were grown in LB medium at 37 °C for 1 h, and then plated on LB agar medium with 50 µg/ml kanamycin, followed by incubation overnight at the same temperature. A single colony was picked from the agar plate to inoculate 500 ml of Terrific Broth (TB) medium supplied with 50 µg/ml kanamycin. The resulting culture was incubated for 24 h at 30 °C in a LEX-24 Bioreactor (Harbinger Biotechnology & Engineering, Markham, Canada) using compressed air for aeration and mixing. Under the conditions used here, there was a considerable level of basal expression (“promotor leakage”), hence no IPTG induction was necessary.

The cells were harvested by centrifugation (6000×*g* for 10 min) at 4 °C, using a Beckman Coulter centrifuge (Brea, CA, USA), and then subjected to periplasmic extraction by osmotic shock as described previously [53]. The periplasmic extracts were sterilized by filtration through a sterile 0.22 µm syringe filter (Sarstedt, Nümbrecht, Germany) and stored at 4 °C prior to purification of the LPMO.

### Protein purification

AA10_07 was purified from a periplasmic extract using ion-exchange chromatography with a HiTrap™ DEAE Sepharose FF 5 ml column (GE Healthcare, Chicago, USA). The enzyme was eluted using a linear gradient of NaCl (0–500 mM) in the starting buffer, which was 50 mM Tris-HCl, pH 7.5. Chromatography fractions were analysed by SDS-PAGE (Bio-Rad, Hercules, California, USA). Fractions containing purified enzyme were pooled and concentrated using Vivaspin ultrafiltration tubes with a molecular weight cut-off of 10 kDa (Sartorius, Göttingen, Germany). The concentrated preparations were subjected to size-exclusion chromatography using a HiLoad 16/60 Superdex 75 column (GE Healthcare, Chicago, USA), with 50 mM Tris-HCl, pH 7.5, containing 200 mM NaCl, which resulted in electrophoretically pure LPMO samples. Protein concentrations were determined by UV–Vis spectroscopy (A_280_) using the theoretical extinction coefficient of AA10_07 (assuming that all pairs of Cys residues form disulphide bonds), as calculated with the ProtParam tool [54].

Copper-saturated enzyme was prepared by co-incubating the purified LPMO with Cu(II)SO_4_ at a 1:3 molar ratio for 30 min, at room temperature in 50 mM Tris-HCl, pH 7.5, containing 200 mM NaCl. Excess copper was removed from the preparation by size-exclusion chromatography on a HiLoad 16/60 Superdex 75 column (as described above) or using a PD MidiTrap G-25 desalting column (GE Healthcare, Chicago, USA) equilibrated with 50 mM sodium phosphate buffer pH 6.0. To avoid contamination with free copper, the sample size in the desalting step with PD MidiTrap G-25 columns was only 350 µl and only the first 1 ml of the eluate (instead of 1.5 ml recommended by the manufacturer) was used in further experiments. In case a HiLoad 16/60 Superdex 75 column was used, subsequent to the gel filtration step, the buffer was exchanged to 50 mM sodium phosphate buffer pH 6.0 using a PD MidiTrap G-25 desalting column. The purified protein samples were stored at 4 °C until further use.

### Assessment of copper content

The total copper content of selected protein and cellulose samples was determined by inductively coupled plasma mass spectrometry (ICP-MS) using a tandem quadrupole 8800 ICP-QQQ machine (Agilent Technologies), equipped with a collision/reaction cell. The samples were mixed with 70% ultrapure nitric acid and a multi-element internal ICP-MS standard (Inorganic Ventures, Christiansburg, VA, USA). All samples were then autoclaved at 121 °C in sealed tubes for 30 min using saturated steam under pressure. Tubes were allowed to cool, and the solutions were diluted with deionized 18.2 MΩ water to 5% (v/v) nitric acid. The ICP-MS instrument was operated in single quadrupole mode using helium as a collision gas to minimize diatomic interferences from plasma or sample. A control standard (Inorganic Ventures, Christiansburg, VA, USA) was analysed in between the protein samples to check and compensate for instrument drift. Calibration curves were prepared prior to protein analysis and the copper concentration was determined according to these curves using Indium as internal standard.

As another method for assessing free copper in enzyme preparations (and its effect on the LPMO reaction), a small volume (≤ 200 µl) of LPMO stock solution was subjected to ultrafiltration using a 3 kDa MWCO 1.5 ml ultrafiltration tube (VWR International, Radnor, PA, USA) and centrifugation at 10,000*g* and room temperature for about 3 min. The protein-free filtrate will contain the same concentration of free copper as the LPMO containing retentate. Filtrates and retentates were collected and used in hydrogen peroxide production experiments (see below).

### Hydrogen peroxide production

H_2_O_2_ production assays were based on the approach previously described by Kittl et al. [13]. 90 µl sample solutions containing LPMO, horse radish peroxidase (HRP) and Amplex Red in 50 mM sodium phosphate buffer pH 6.0 were pre-incubated in a 96-well microtiter plate for 5 min at 30 °C. The reactions were initiated by the addition of 10 µl of a reductant stock solution. The final concentrations of LPMO, HRP, Amplex Red and reductant were 3 µM, 5 U/ml, 100 µM and 50 µM (or 1 mM), respectively. For each experiment, control reactions were set up by substituting the LPMO with the same volume of water and/or with the same volume of a protein-free sample (“filtrate”), produced by ultrafiltration of the LPMO stock solution, as described above.

The formation of hydrogen peroxide was monitored by recording the optical absorbance of resorufin (the product generated from Amplex Red by HRP) at 563 nm over time, at 30 °C, using a Varioscan LUX plate reader (Thermo Fisher Scientific, Waltham, MA, USA). H_2_O_2_ standard solutions were prepared in 50 mM sodium phosphate buffer pH 6.0 and supplied with 5 U/ml HRP and 100 µM Amplex Red to generate a standard curve. Apparent H_2_O_2_ production rates were derived from the initial linear parts of the resorufin production curves.

Hydrogen peroxide production in reactions containing free copper and a reductant (gallic acid or ascorbic acid) was assessed in the same manner as described above, using various concentrations of Cu(II)SO_4_.

### LPMO reactions with Avicel

The LPMO activity on microcrystalline cellulose was studied by setting up reactions with varying amounts of enzyme (0.05–8 µM) and 1% (w/v) Avicel in 50 mM sodium phosphate buffer pH 6.0, supplied with 1 mM reductant (gallic acid or ascorbic acid) and varying amounts of CuSO_4_ or H_2_O_2_. The reactions were carried out in a thermomixer (30 °C, 900 RPM). Note that LPMO reactions in the presence of gallic acid were set up using a 100 mM stock solution of gallic acid in DMSO. Thus, 1% (v/v) DMSO was introduced to the reaction mixtures. Control experiments were performed to confirm that 1% (v/v) DMSO does not have a significant impact on the LPMO reaction (Additional file 1: Fig S6).

100 µl aliquots were taken at various time points, and the reactions were stopped by immediately separating the enzyme and soluble products from the insoluble substrate by filtration using a 96-well filter plate (Millipore, Burlington, MA, USA), after which the filtrates were stored at − 20 °C. Note that the product profile of AA10_07 (see Fig. 2b) shows a range of shorter to longer oxidized products (DP2-7ox) with DP6ox being the most prominent product. If AA10_07 was capable of degrading soluble cello-oligosaccharides, accumulation of shorter products would be expected, but this is not the case. Thus, the reactions were considered quenched after filtration. For qualitative product analysis, the filtrates were subjected to analytical chromatography without any additional pre-treatment procedures. Prior to quantitative product analysis, the filtrates were incubated at 37 °C, overnight with 1 µM in-house produced recombinant *Thermobifida fusca* GH6 endoglucanase (*Tf*Cel6A; [55]) to convert oxidized cello-oligosaccharides to a mixture of oxidized dimers and trimers only.

### Product analysis by HPAEC-PAD

Cellulose degradation products were analysed by high-performance anion-exchange chromatography with pulsed amperometric detection (HPAEC-PAD) using a Dionex ICS5000 system (Thermo Scientific, San Jose, CA, USA) equipped with a CarboPac PA200 analytical column. A stepwise gradient with an increasing amount of eluent B (eluent B: 0.1 M NaOH and 1 M NaOAc; eluent A: 0.1 M NaOH) was applied according to the following program: 0–5.5% B over 3 min, 5.5–15% B over 6 min, 15–100% B over 11 min, 100–0% B over 6 s, 0% B over 6 min. The flow rate was 0.5 ml/min. Chromeleon 7.0 software was used for data analysis. C1-oxidized cello-oligosaccharide standards with a degree of polymerization of two and three (DP2, DP3) were prepared in-house as described before [41, 56].

### Product analysis by MALDI-ToF MS

Products of Avicel degradation were identified using a matrix-assisted laser desorption/ionization time-of-flight (MALDI-ToF) UltrafleXtreme mass spectrometer (Bruker Daltonics GmbH, Bremen, Germany). 1 μl of the LPMO reaction mixture was mixed with 2 μl of a matrix solution (9 mg/ml 2,5-dihydrooxybenzoic acid) on a MTP 384 ground steel target plate (Bruker Daltonics). The plate was air-dried, and the spectral data were acquired using Bruker flexControl software, as described previously [1].

## Supplementary Information

**Additional file 1: Fig S1.** Cellulose solubilization by two batches of AA10_07. **Fig S2.** Underestimation of H_2_O_2_ in the presence of ascorbic acid or gallic acid. **Fig S3.** Cellulose solubilization by increasing amounts of AA10_07 in the presence of ascorbic acid or gallic acid. **Fig S4.** Cellulose solubilization by 1 μM AA10_07 in the presence of protein-free filtrates. **Fig S5.** Comparing the activity of two different batches of ScLPMO10C. **Fig S6.** The effect of DMSO on AA10_07 reactions with cellulose.

## Data Availability

Data supporting the findings of this work are available within the paper and its Additional information file and from the corresponding author upon reasonable request.
