# Supplementary material for: Unraveling the roles of the reductant and free copper ions in LPMO kinetics
Source: Biotechnol Biofuels. 2021 Jan 21;14:28. doi: 10.1186/s13068-021-01879-0 (PMC7818938; doi:10.1186/s13068-021-01879-0)
Supplement: Supplementary file 1 — Additional file 1: Fig S1. Cellulose solubilization by two batches of AA10_07. Fig S2. Underestimation of H2O2 in the presence of ascorbic acid or gallic acid. Fig S3. Cellulose solubilization by increasing amounts of AA10_07 in the presence of ascorbic acid or gallic acid. Fig S4. Cellulose solubilization by 1 μM AA10_07 in the presence of protein-free filtrates. Fig S5. Comparing the activity of two different batches of ScLPMO10C. Fig S6. The effect of DMSO on AA10_07 reactions with cellulose. [file 13068_2021_1879_MOESM1_ESM.pdf]

Unraveling the roles of the reductant and free copper ions in LPMO kinetics

Anton A. Stepnov[1], Zarah Forsberg[1], Morten Sørlie[1], Giang-Son Nguyen[2], Alexander Wentzel[2], Åsmund K. Røhr[1] and Vincent G.H. Eijsink[1*]

[1] Faculty of Chemistry, Biotechnology and Food Science, NMBU - Norwegian University of Life Sciences, Ås, Norway

[2]Department of Biotechnology and Nanomedicine, SINTEF Industry, Trondheim, Norway

[*] corresponding author, vincent.eijsink@nmbu.no

Additional file 1

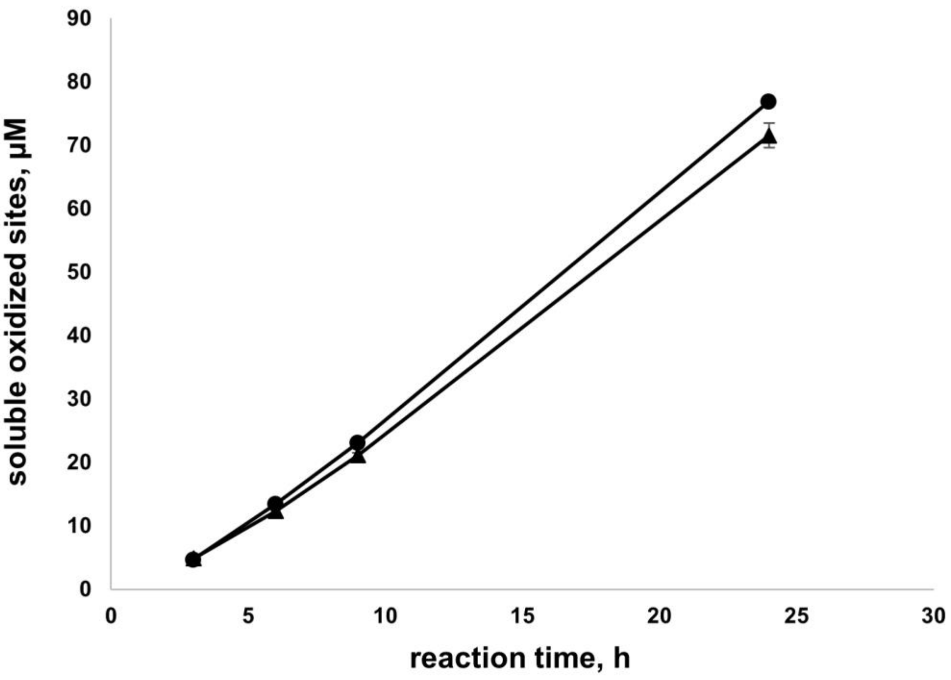

Fig S1. Cellulose solubilization by two batches of AA10_07. The figure shows progress curves obtained in LPMO reactions (1 µM AA10_07 in 50 mM sodium phosphate buffer, pH 6.0, 30 °C) with 1% (w/v) Avicel using desalted (circular markers) and SEC-treated (triangular markers) batches of AA10_07. For reduction, 1 mM ascorbic acid was present in the reaction mixtures. Error bars indicate standard deviations between triplicates. Prior to quantification of oxidized sites, soluble LPMO products of varying lengths were converted to a mixture of oxidized dimers and trimers by treatment with a cellulase.

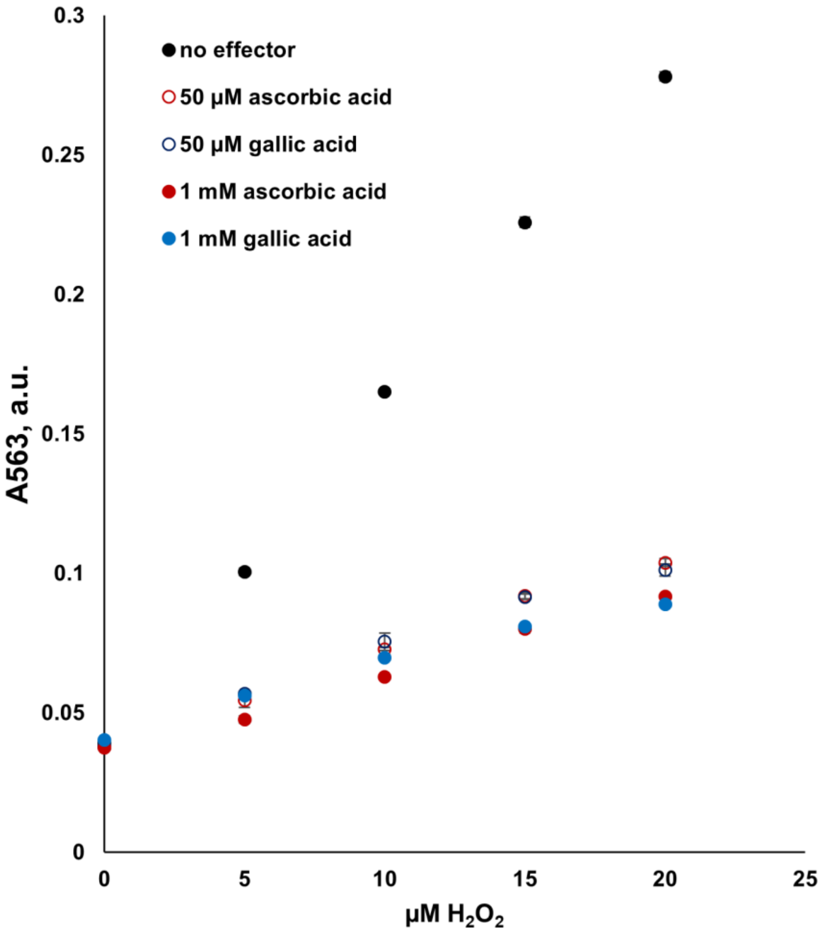

Fig S2. Underestimation of H_2O_2 in the presence of ascorbic acid or gallic acid. The figure shows HRP/Amplex Red results, obtained using various amounts of hydrogen peroxide in the presence and in the absence of 50 µM or 1 mM reductants. Note that both ascorbic acid and gallic acid supress the signal, i.e., the H_2O_2-fueled conversion of Amplex Red to the red-fluorescent oxidation product, resorufin, to a similar extent. Error bars indicate standard deviations between triplicates.

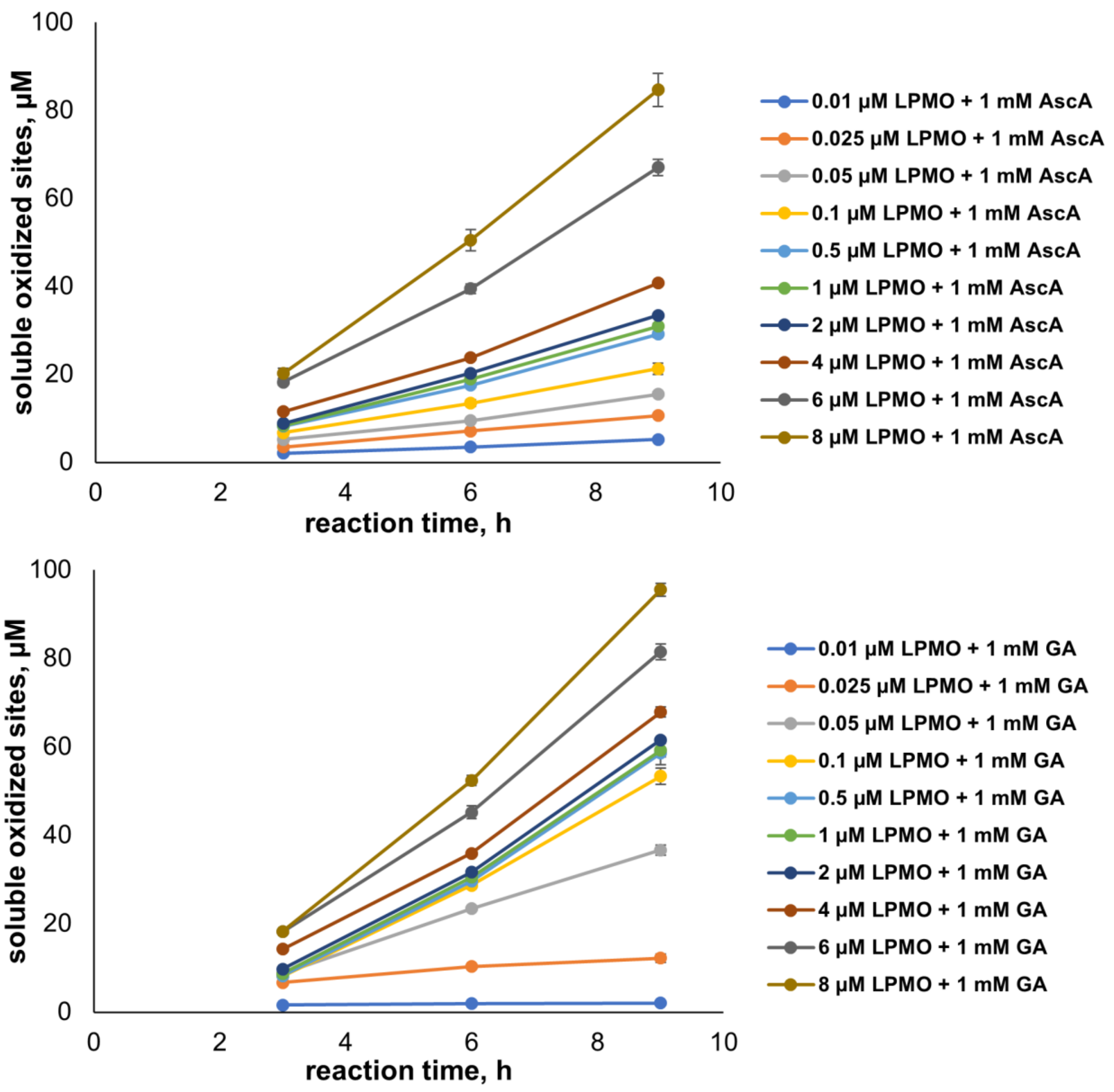

Fig S3. Cellulose solubilization by increasing amounts of AA10_07 in the presence of ascorbic acid or gallic acid. The figure shows progress curves obtained in LPMO reactions (in 50 mM sodium phosphate buffer, pH 6.0, 30 °C) with 1% (w/v) Avicel using 0.01 µM – 8 µM AA10_07. For reduction, 1 mM ascorbic acid or gallic acid was present in the reaction mixtures. AscA, ascorbic acid; GA, gallic acid. Error bars indicate standard deviations between triplicates. Product accumulation was not observed in control reactions with 1% (w/v) Avicel and no enzyme present. The 9 h end points were used to generate Fig. 7 in the main manuscript.

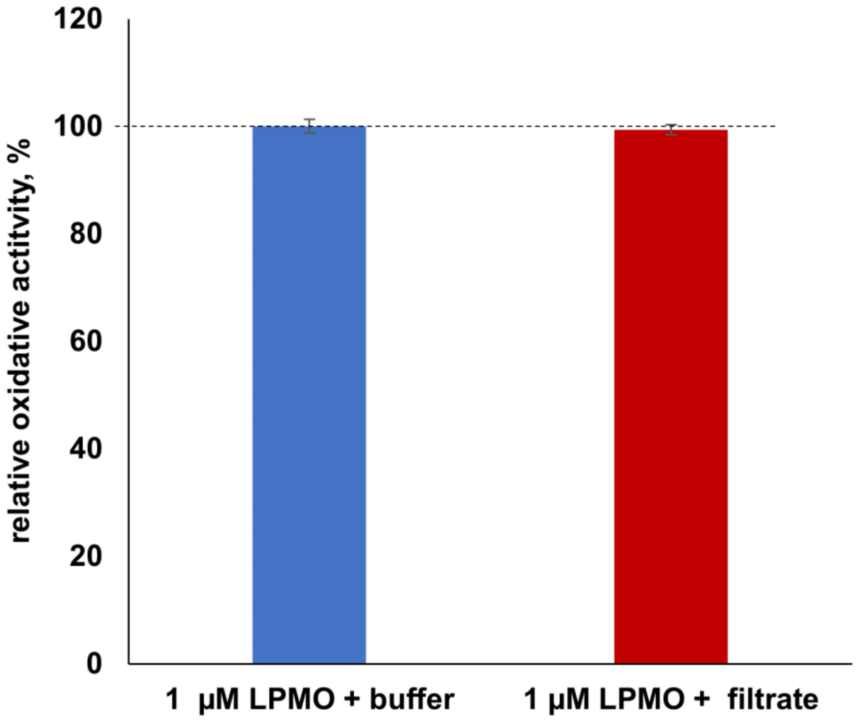

Fig S4. Cellulose solubilization by 1 µM AA10_07 in the presence of protein-free filtrates. The figure shows the activity of 1 µM AA10_07 in 6 hours reactions with 1% (w/v) Avicel in the absence or presence of protein-free solution obtained by ultra-filtration of the enzyme preparation. This control solution contained the same amount of free (unbound) copper as the LPMO stock solution used in this study. The control reaction (red bar) was started by mixing 5.2 µL of the enzyme stock solution with 41.6 µL (8 times more volume) of protein-free filtrate. In the reference reaction (blue bar), the protein-free filtrate was substituted by the same volume of 50 mM sodium phosphate buffer, pH 6.0. Note that due to addition of the filtrate, the control reaction with 1 µM LPMO (red bar) will contain the same concentration of free copper as a reaction with 9 µM LPMO. The graph shows that, despite the nine-fold increase in the (potentially very low) free copper concentration, the experiment with the filtrate did not show any boost to LPMO activity, compared to the reference reaction (blue bar; 100 %). This result indicates that free copper levels in the AA10_07 preparation were too low to bias the results of the LPMO dose-response experiments shown in Fig. 7 & S3. Reaction conditions: 57 mM sodium phosphate buffer, pH 6.0, 30 °C. 1 mM ascorbic acid was used as a reductant. Error bars indicate standard deviations between triplicates.

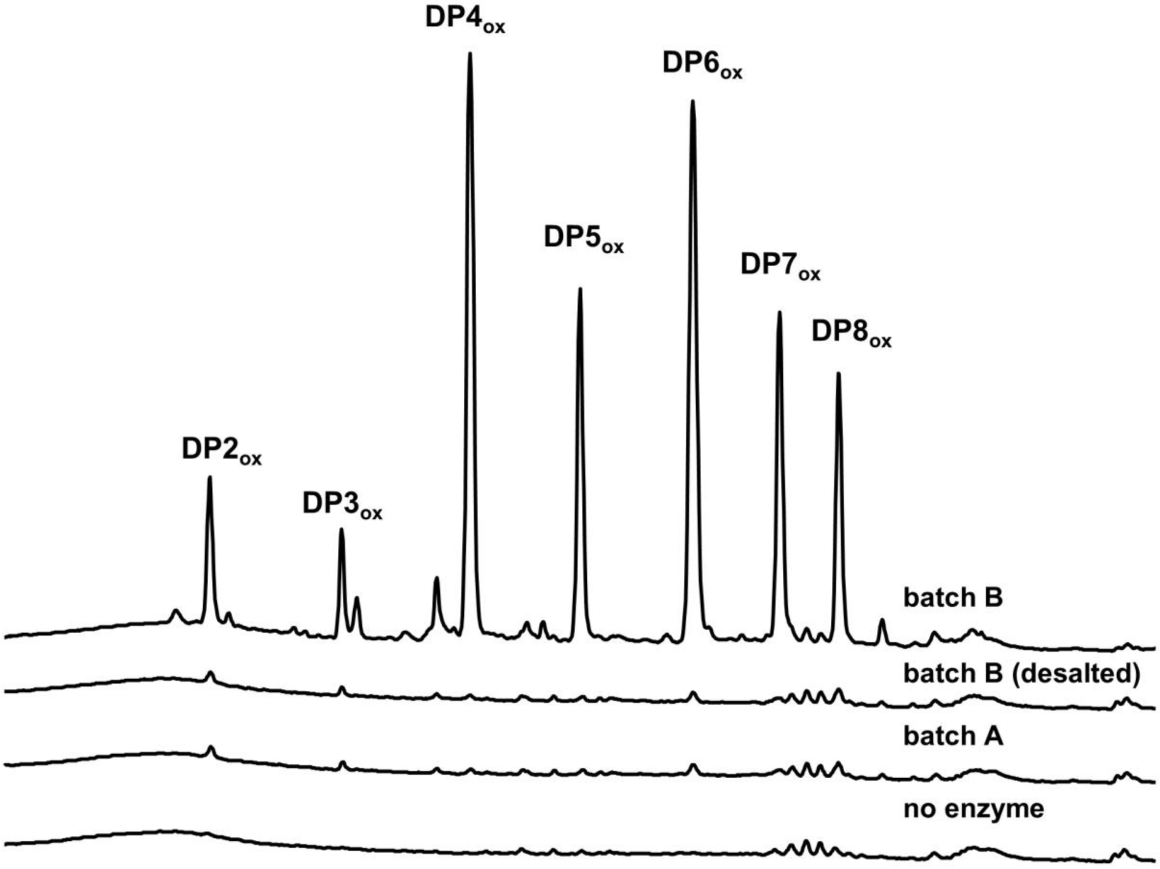

Fig S5. Comparing the activity of two different batches of *Sc*LPMO10C. The figure shows HPAEC-PAD chromatograms of products released by 1 µM *Sc*LPMO10C in 1 hour reactions (50 mM sodium phosphate buffer, pH 6.0, 30 °C) with 1 % (w/v) Avicel, supplied with 1 mM ascorbic acid. Batch A, *Sc*LPMO10C preparation obtained in this study; batch B, previously produced *Sc*LPMO10C sample, without or with an additional desalting step, as indicated. Oxidized product peaks were annotated based on previously published product profiles for *Sc*LPMO10C [2, 12, 42].

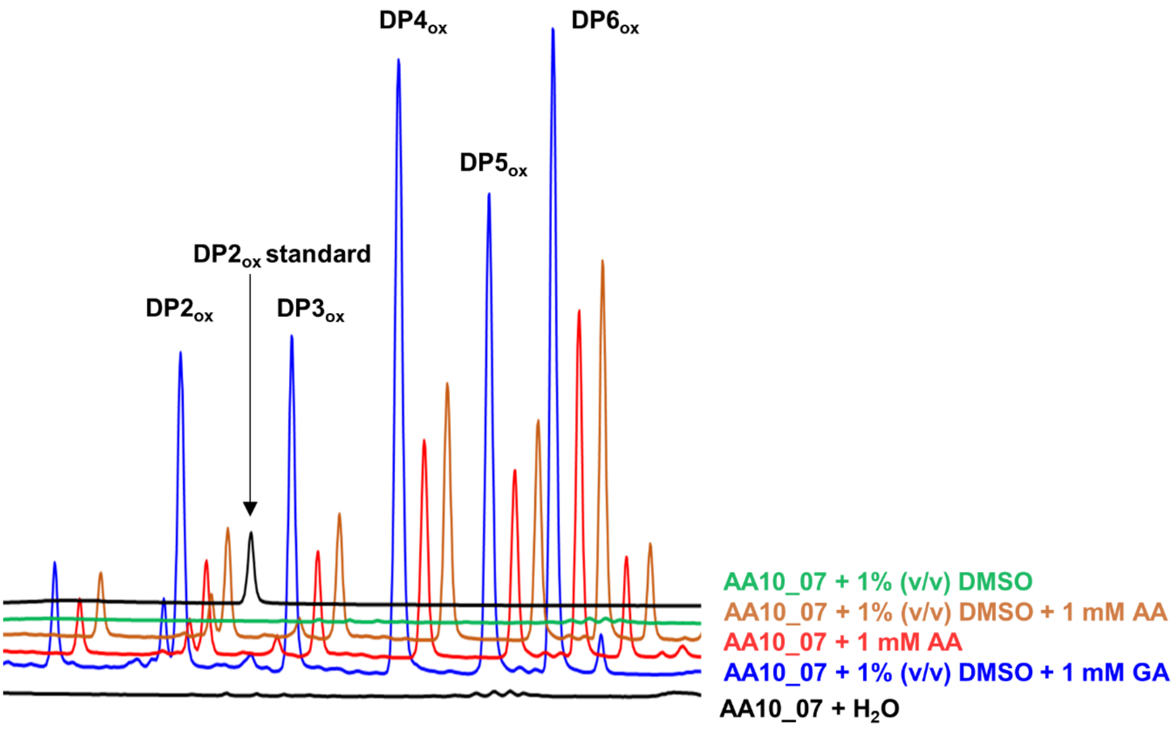

Fig S6. The effect of DMSO on AA10_07 reactions with cellulose. The figure shows HPAEC-PAD chromatograms of oxidized products released by AA10_07 in 24 hour reactions (1 µM LPMO in 50 mM sodium phosphate buffer, pH 6.0, 30 °C) with 1 % (w/v) Avicel in the presence of 1 mM reductant and/or 1% (v/v) DMSO. Note that the control experiment, performed at the same conditions, indicates that 1% (v/v) DMSO alone is not able to act as LPMO reductant. The oxidized products peaks are annotated based on retention times relative to the GlcGlc1A ($DP2_{ox}$) standard.
